# Potential enhanced association between obstructive lung disease and history of depression in patients with diabetes

**Published:** 2021

**Authors:** Maria E Ramos-Nino, Charles D MacLean, Benjamin Littenberg

**Affiliations:** 1 St. George’s University, Department of Microbiology, Immunology, and Pharmacology, Grenada, West Indies; 2 Department of Pathology and Laboratory Medicine, University of Vermont 05401, Burlington, Vermont, USA; 3 Department of Medicine, University of Vermont, Burlington, Vermont 05401, USA

**Keywords:** Obstructive lung disease, Asthma, COPD, Depression, Diabetes

## Abstract

**Background:**

Depression is one of the most common comorbidities of chronic diseases including diabetes and obstructive lung diseases (emphysema, chronic bronchitis, and asthma). Obstructive lung diseases and depression have few symptoms in common. However, they are both common in adults and associated with chronic inflammation. It is not clear if their coappearance in diabetic patients is coincidental or associated beyond that expected by chance.

**Methods:**

A total of 1,003 adults with diabetes in community practice settings were interviewed at home at the time of their enrolment into the Vermont Diabetes Information System, a clinical decision support program. Patients self-reported their personal and clinical characteristics, including any obstructive lung disease. Laboratory data were obtained directly from the clinical laboratory, and current medications were obtained by direct observation of medication containers. We performed a cross-sectional analysis of the interviewed subjects to assess a possible association between the prevalence of obstructive lung disease and depression.

**Results:**

In a multivariate logistic regression model, obstructive lung disease was significantly associated with depression even after correcting for gender, obesity (≥30 kg/m^2^), high comorbidities (>2), low annual income (<$30,000/ year), cigarette smoking, alcohol problems, and education level (odds ratio=1.83; 95% confidence interval 1.27, 2.62; *P* <0.01).

**Conclusion:**

These data suggest a potential enhanced association between obstructive lung disease and depression in patients with diabetes. Future studies are needed to identify if inflammation is implicated in this association as a common denominator.

## Background

Chronic diseases have increased in prevalence over the past decades in America [1,2]. Recent evidence suggests a link between inflammation and several chronic conditions including diabetes, cardiovascular disease, cancer, rheumatoid arthritis, inflammatory bowel disease, asthma, and chronic obstructive lung disease [3]. Together, chronic inflammatory diseases are the most significant cause of death worldwide [1].

Causes of systemic chronic inflammation include chronic infections, physical inactivity, obesity, intestinal dysbiosis, diet, psychological stress, exposure to xenobiotics such as tobacco smoking, and others [4]. The chronic inflammatory process activated by these triggers may lead to type 2 diabetes, steatohepatitis, cardiovascular disease, cancer, depression, autoimmune diseases, and neurodegenerative diseases (reviewed in [4]).

We aim to explore the association between two apparently independent chronic diseases, obstructive lung disease [asthma, and chronic obstructive pulmonary disease (COPD)] and depression, in a diabetic population. Symptoms of depression and anxiety have been reported before in individuals with obstructive lung disease as well as those with diabetes [5–9]. Depression and anxiety can impact quality of life and disease burden [6,10], as well as the development of complications and higher mortality rates [7,9,10]. Whether inflammation is the cause, or an effect of depression is not clear. Many studies have found elevated inflammatory cytokines and acute-phase proteins in patients with depression [11,12]. Chronic exposure to inflammation is thought to induce changes in neurotransmitters and neurocircuits that lead to depressive symptoms and that may interfere with treatment [11,13–15]. However, contradictory findings regarding the association between depression and inflammation can also be found in the literature [16].

## Methods

This study is part of a larger project, the Vermont Diabetes Information System, a study of 7,412 adults with diabetes in primary care practices [17]. The subjects comprised all diabetic adults in 64 practices in Vermont and adjacent New York. A field survey was completed at study baseline with a subsample of subjects. Patients’ names were randomly sorted, and patients were contacted by telephone until a sample of approximately 15% of patients from each practice agreed to participate in the field survey to give a sample of 1,007 at the time of analysis. Four patients were dropped from the analysis due to incomplete information, leaving a final sample of 1,003.

Subjects completed a questionnaire at home and then were visited by a trained research assistant who reviewed the questionnaire responses, assisted the subject with any missing or unacceptable responses, reviewed the subject’s medications by direct examination of all medication containers, and measured their height and weight using a portable stadiometer and scale. Race, education, income, marital status, functional status, smoking, alcohol consumption, and comorbid conditions were obtained by questionnaire. To determine comorbidity, we used a modification of the Self-Administered Comorbidity Questionnaire [18] in which we asked each patient to indicate whether they had had the following conditions: heart attack, heart failure, peripheral arterial disease, stroke, dementia, rheumatic disease, peptic ulcer, cirrhosis, paralysis, renal insufficiency, diabetic vascular complications, AIDS/HIV, and depression. The primary outcome variable, presence of obstructive lung disease, was the patient’s response to the question “Do you have asthma, emphysema, or chronic bronchitis?”. The primary predictor variable, history of depression, was determined by the patient’s responses to the following question: “Have you had depression?”

Most laboratory data were obtained from the patients’ local clinical laboratories, which all used the same Diabetes Control and Complications Trial/Epidemiology of Diabetes Interventions and Complications high performance, liquid chromatography (HPLC) method for the determination of glycosylated hemoglobin (A1C). Less than 1% of A1C tests were performed using the Bayer DCA 2000 immunoassay point of care instrument, which compares favorably with the HPLC method [19].

The research protocol was approved by the Committee on Human Research of the University of Vermont. The interviewed subjects provided written informed consent. The full study protocol and variables and the medication profiles of the subjects have been previously reported [17,20].

### Statistical approach

We used logistic regression to assess the univariate relationship of obstructive lung disease as the outcome variable with history of depression as the predictor. We then adjusted for possible confounding by social and clinical factors. Potential confounders tested were gender (male/female), age (years), race (White/other), obesity (body mass index (BMI) ≥30 kg/m^2^), high comorbidities (>2 excluding diabetes, obstructive lung disease, and depression), glycosylated hemoglobin level (A1C; %), insulin use (yes/no), duration of diabetes in years, self-reported history of alcohol problems (yes/no), cigarette smoking (yes/no), low income (<$30,000 per year), and level of education (High School graduate or more). To reduce the number of variables in the final model, we excluded potential confounders that were associated with the outcome in univariate analyses with *P* >0.15. Such a weak association implies that the variable is unlikely to be a confounder. We used Stata/SE v.16 (StataCorp, College Station, TX, USA) for all analyses.

## Results

The general characteristics of the study population are described in Table 1. The study population was representative of adults with diabetes in primary care practices in northern New England, USA. Because many of the subjects were over retirement age or suffered disabilities from chronic conditions, their income was lower than that of healthy younger Americans. The number of patients with obstructive lung disease in this population was 203 (20.2%). A total of 351 (35.0%) of the subjects had a history of depression (Table 1).

Table 2 presents univariate associations between obstructive lung disease and the other study variables that had the potential of being significantly associated with obstructive lung disease prevalence (Table 2).

Next, potential confounding variables associated with obstructive lung disease with P<0.15 were included in a logistic regression model using obstructive lung disease as the outcome (Table 3). This model showed a significant association between obstructive lung disease and depression (odds ratio (OR)=1.83; confidence interval (CI) (1.28-2.62); P<0.01).

## Discussion

The prevalence of depression in diabetic patients is often higher than that in the general population, and depression accounts for a substantial part of the psychosocial burden of these disorders [21]. Although treatments can be effective, adjustments may be needed for patients with comorbidities. In addition, symptoms or treatments of comorbidities may interfere with the treatment of depression, and symptoms of depression may decrease adherence to treatment in these patients. Our data indicate that 20.2% of this cohort had chronic obstructive lung disease and 35% of the population had a history of depression. Furthermore, patients with chronic obstructive lung disease in this cohort had a higher prevalence of depression history (51.2%) compared to the population without obstructive lung disease (30.9%). These findings demonstrate a significant enhanced association between obstructive lung disease and depression in this diabetic cohort. Although it is unclear if depression causes lung disease or the reverse, we hypothesize that both are caused by a third factor: chronic inflammation, which is present in obstructive lung disease, diabetes, and depression [5–7,12]. Although social factors clearly play a role in these relationships, those we could measure were unlikely to explain the entire phenomenon.

One of the strengths of this study is that the interviewed subjects were a randomly selected subset of a large population of patients receiving care in New England, which makes them likely to be representative of other primary care patients in the US.

This study does, however, have several limitations, including self-report of obstructive lung disease, lack of confirmation of obstructive lung disease and depression diagnoses, inability to distinguish between asthma and COPD patients, and lack of information on the time relation between the onset of obstructive lung disease and the onset of depression. As in any cross-sectional study, unmeasured confounders could be responsible for the apparent associations found.

## Conclusion

Our findings suggest that the presence of obstructive lung disease may enhance depression in patients with diabetes. The diagnosis of depression in diabetic patients with obstructive lung diseases is important as it may be related to disease control and prognosis. Further research is needed to identify the mechanisms involved.

## Figures and Tables

**Table 1:** Baseline characteristics of 1,003 adults with diabetes.

Characteristic	N (%) or mean (sd)
Age, years	64.8 (12.0)
Gender: Men	457 (45.6%)
Gender: Women	546 (54.4%)
White race	973 (97.3%)
Median income ($/year)	15,000-29,999
Low annual income (<$30,000)	548 (59.1%)
Level of education (High School graduate or higher)	762 (75.7%)
Body mass index (BMI) kg/m^2^	33.8 (7.4)
Obese (BMI > 30 kg/m^2^)	666 (67.3%)
Glycosylated hemoglobin A1C %	7.1 (1.3)
Insulin use	186 (18.5%)
Duration of diabetes, years	10.2 (10.3)
Obstructive lung disease prevalence	**203 (20.2%)**
Depression	**351 (35.0%)**
High number of comorbidities (>2)	613 (60.9%)
Alcohol problem	78 (7.9%)
Cigarette smoking	170 (17.0%)

sd: standard deviation; n: number of subjects with the characteristic.

**Table 2:** Univariate associations between obstructive lung disease and other patient characteristics.

Characteristic	Obstructive lung disease patients	Non-obstructive lung disease patients	OR	P
Number of subjects	203	800		
**Depression**	**51.2%**	**30.9%**	**2.35**	**<0.01**
Age, years	64.3 (11.4)	64.9 (12.1)	1.00	0.54
Male	33.5%	48.6%	0.53	<0.01
White race	96.1%	97.6%	0.60	0.23
Low annual income	75.7%	54.8%	2.57	<0.01
High School graduate	64.5%	78.4%	0.50	<0.01
Obese (BMI>30 kg/m^2^)	77.0%	64.8%	1.82	<0.01
A1C, mg %	7.2 (1.3)	7.1 (1.3)	1.03	0.67
Insulin use	27.2%	22.8%	1.27	0.20
Duration of diabetes, years	11.1 (10.6)	10.0 (10.3)	1.01	0.20
High comorbidities (>2)	73.4%	57.5%	2.04	<0.01
Alcohol problem	12.1%	6.8%	1.88	0.02
Cigarette smoking	24.3%	15.1%	1.79	<0.01

Each cell contains either % or mean (standard deviation).

**Table 3:** Multivariate logistic regression: obstructive lung disease vs. depression and potential confounders (N=903)

Characteristic	OR	*P*	95% CI
**Depression**	**1.83**	**<0.01**	**1.28, 2.62**
Gender (male)	0.52	<0.01	0.36, 0.75
Low annual income	1.78	0.01	1.18, 2.69
High School graduate	0.58	0.01	0.40, 0.86
Obesity	1.63	0.02	1.09, 2.44
High comorbidities	1.77	<0.01	1.21, 2.58
Alcohol problem	1.56	0.13	0.88, 2.77
Cigarette smoking	1.43	0.10	0.93, 2.18

## Data Availability

All data analyzed during this study are included in this published article.

## Abbreviations:

VDIS: Vermont Diabetes Information System
BMI: Body Mass Index
COPD: Chronic Obstructive Pulmonary Disease
A1C: Glycosylated hemoglobin
CI: Confidence Interval
SD: Standard Deviation
N: Number of subjects with the characteristic
OR: Odds Ratio
